# Multicenter registry of pediatric inflammatory bowel disease from a developing country

**DOI:** 10.1186/s12887-024-04698-y

**Published:** 2024-04-01

**Authors:** Pornthep Tanpowpong, Settapong Jitwongwai, Teera Kijmassuwan, Hansa Sriphongphankul, Seksit Osatakul, Alisara Damrongmanee, Nuthapong Ukarapol, Suporn Treepongkaruna

**Affiliations:** 1 Division of Gastroenterology, Department of Pediatrics, Faculty of Medicine Ramathibodi Hospital, Mahidol University, Bangkok, Thailand; 2 Department of Pediatrics, Faculty of Medicine Siriraj Hospital, Mahidol University, Bangkok, Thailand; 3 Department of Pediatrics, Faculty of Medicine, Prince of Songkla University, Songkhla, Thailand; 4 Department of Pediatrics, Faculty of Medicine, Chiang Mai University, Chiang Mai, Thailand

**Keywords:** Diagnostic delay, Diarrhea, Growth failure, Perianal fistula, Steroids

## Abstract

**Background:**

Despite the rising incidence of pediatric inflammatory bowel disease (PIBD) globally, multicenter collaborative studies of PIBD children among developing countries remain sparse. We therefore aimed to define the initial presentation and short-term outcomes of Thai children with PIBD from a multicenter registry.

**Methods:**

Four teaching hospitals participated in this study. A diagnosis of PIBD requires gastrointestinal endoscopy and histopathology in children aged < 19 years. Besides demographics, we collected clinical information and treatment with the data at 1-year follow up.

**Results:**

We included 35 Crohn’s disease (CD), one IBD-unclassified, and 36 ulcerative colitis (UC) children (total *n* = 72 with 60.6% males). The mean age at diagnosis was 7.9 years (SD 4.1) with 38% being very early onset IBD (VEO-IBD). When compared with UC, the CD children were more likely to exhibit fever (42.3 vs. 13.9%), weight loss/failure to thrive (68.6 vs. 33.3%), and hypoalbuminemia (62.9 vs. 36.1%) but less likely to have bloody stools (51.4 vs. 91.7%) (all *P* < 0.05). No significant differences in demographics, clinical data and medications used with regards to VEO-IBD status. At 1 year after diagnosis (*n* = 62), 30.7% failed to enter clinical remission and 43.7% remained on systemic corticosteroids. Diarrhea (OR 9.32) and weight issues (OR 4.92) at presentation were independent predictors of failure to enter clinical remission; and females (OR 3.08) and CD (vs. UC) (OR 3.03) were predictors of corticosteroids use at 1-year follow-up.

**Conclusions:**

A high proportion of VEOIBD is noted, and CD was more likely to present with significant inflammatory burden. Diarrhea and weight issues at presentation were independent predictors of failure to enter clinical remission; and females and CD (vs. UC) were predictors of corticosteroids use at 1-year follow-up.

## Introduction

Pediatric-onset inflammatory bowel disease (PIBD) is a chronic idiopathic inflammatory disorder of the gastrointestinal (GI) tract that can present early in life [1]. The disease can be divided into 3 subtypes: Crohn’s disease (CD), ulcerative colitis (UC) and inflammatory bowel disease-unclassified (IBD-U) [2]. A 2022 systematic review reported a rising incidence and prevalence of PIBD in 84% and 100% among 37 and 7 included studies, respectively [3]. The incidence and prevalence of PIBD between geographic regions are different as being highest in the Northern Europe and North America and lowest in the Middle East and Asia.

Data from the multinational collaborative studies to define the characteristics and courses of PIBD children in 6 Asian countries (Malaysia, Philippines, Singapore, Sri Lanka, Taiwan, and Thailand) have been reported [4]. One of the main findings was that PIBD children from the cohort were diagnosed earlier than children residing in Europe and Israel from the EUROKIDS registry (7.7 vs. 12.5 years in CD and 10.2 vs. 11.6 years in UC, respectively) [5, 6]. Even with the increasing trend, time from the disease onset to the time of established IBD diagnosis in children and adults may take several months demonstrated in three recent systematic reviews [7–9]. Longer diagnostic delay has been associated with growth faltering, more extensive disease, and a worse response to treatment in children; [8] while a systematic review of 11 studies in 6,164 adults showed that delayed diagnosis was associated with higher chances of developing stricturing and penetrating disease, intestinal surgery in CD as well as colectomy in UC [9]. IBD population in low- to middle-income countries was even diagnosed later than individuals living in the high-income countries [8], as heterogeneities in disease awareness, availability and timeliness of diagnostic evaluation and management processes may be observed between the regions. To the best of our knowledge, only one report on PIBD in Thailand was published in 2006 [10]. In addition, only one center in Thailand participated in the recent Asian PIBD study group [4]. 

To stimulate disease awareness within similar health care settings, we therefore conducted a multicenter study to define the clinical characteristics, diagnostic delay, IBD-related investigations, management and short-term outcomes of PIBD in Thailand.

## Methods

The national collaborative network was initiated in 2020 among the 4 institutions (2 in the central region, 1 in the northern region and 1 in the southern region). All institutions are tertiary care teaching hospitals and have university-based settings with referrals from the hospitals within the region. A consensus was established amongst the network members with regard to standardized data collection, and the study was conducted between March 2021 and December 2022. Retrospective cases were PIBD children diagnosed between January 2014-December 2019 and cases diagnosed from January 2020 to August 2022 were collected prospectively. All diagnosed cases had endoscopy and histology findings consistent with PIBD before aged 19 years. Baseline demographic and clinical data were anonymized throughout the process of data collection, data entry, and biostatistical analysis. Institutional Review Boards/Ethics Committees of each institution approved the study with formal data/material transfer agreements between institutions.

Collected data included patient demographics, time from disease onset to PIBD diagnosis, diagnostic evaluation, and disease characteristics based on clinical examination, laboratory, and endoscopy with histopathology. Information on the management and disease outcomes such as 1-year clinical remission and systemic corticosteroid use were also collected. The diagnosis and classification of PIBD was classified into either disease subtypes (CD, UC, or IBD-U) was determined by each participating center based on the revised Porto criteria [2]. The Paris Classification was used to define disease behaviors and phenotypes [11]. The date of IBD diagnosis was the date of diagnostic endoscopy and histopathology. Early onset IBD (EO-IBD) was defined if diagnosed by the age of 10 years and very early onset IBD (VEO-IBD) if diagnosed by age < 6 years. A perianal manifestation was defined if large perianal tags, fistula and/or abscess or collection were noted. We included various clinical and laboratory data within the 3-month period from the date of diagnosis. Anemia for age was defined if hemoglobin < 11 g/dL if aged < 5 years, < 11.5 g/dL if aged 5–11 years, < 12 g/dL in adolescent females and < 13 g/dL in adolescent males. Thrombocytosis was defined if platelet count was > 450,000/cu mm. Erythrocyte sedimentation rate (ESR) of ≥ 20 mm/h and C-reactive protein (CRP) of ≥ 5 mg/L were considered abnormally high. Hypoalbuminemia was defined if serum albumin was < 35 g/L. Disease severity was defined based on the Pediatric Crohn’s Disease Activity Index (PCDAI) [inactive: < 10, mild: 10–30, moderate-severe: >30] [12] and Pediatric Ulcerative Colitis Activity Index (PUCAI) [inactive: <10, mild: 10-<35, moderate: 35-<65, severe ≥ 65] [13]. Clinical remission was defined if either PCDAI or PUCAI was < 10. At 1 year after diagnosis, we also accounted for the information within the range of 3 months (i.e., 9–15 months since the PIBD diagnosis).

### Biostatistical analyses

The analyses were performed using STATA 16.0 (College Station, Texas, USA). Study variables were presented as frequency and percentage, mean with standard deviation (SD), or median with interquartile range (IQR). Categorical variables were compared using a t-test, Mann-Whitney U test, Chi-square test, or Fisher’s exact test. Univariate and multivariate analyses were performed by binary and multiple logistic regression for the interested factors. Forward stepwise selection was performed to choose the variables with *P* < 0.10 to enter the multivariable models. Statistical significance was defined as *P*-value < 0.05.

## Results

During the study period, 72 PIBD children were included in the database with a relatively similar proportion of CD and UC (35 and 36 children, respectively) and one patient with IBD-U. Almost 70% were PIBD cases during the retrospective period (2014–2019) without a significantly increased trend throughout the study period (up to August 2022), with 6–10 newly diagnosed patients per year without significant difference between CD and UC. The mean age at diagnosis was 7.9 years (SD 4.1) with 38% diagnosed before aged 6 years (i.e., VEO-IBD). The age distribution between CD and UC was relatively similar with regard to the proportions of EO-IBD and VEO-IBD (Table 1). The ratio of male to female was more pronounced among CD when compared to UC (2.18:1 vs. 1.12:1, respectively). All except two children were Thais. A mean duration from onset to diagnosis was 9.5 months (SD 13.4).

**Table 1:** Features of IBD children compared between Crohn’s disease vs. ulcerative colitis

Characteristics	IBD phenotypes			*P* value (CD vs. UC)
	Overall, *n* = 72^*^	CD, *n* = 35	UC, *n* = 36	
Age at diagnosis, mean (SD), years Early onset IBD (diagnosed < 10 years), % Very early onset IBD (diagnosed < 6 years), %	7.9 (4.1) 62.5 38.0	8.1 (4.6) 57.1 40.0	7.8 (3.5) 66.7 36.1	0.76 0.41 0.77
Female, %	39.4	31.4	47.2	0.17
Male to female ratio	1.54:1	2.18:1	1.12:1	
Duration from onset to diagnosis, mean (SD), months	9.5 (13.4)	6.9 (10.3)	12.5 (15.6)	0.08
Presenting symptoms and signs, %				
Abdominal pain Diarrhea Bloody stools Fever Pallor Weight loss/failure to thrive Perianal lesions (large perianal skin tags, fistula, abscess)	43.7 69.0 71.8 28.2 38.0 50.7 -	45.7 62.9 51.4 42.3 34.3 68.6 31.4	41.7 75.0 91.7 13.9 41.7 33.3 N/A	0.73 0.27 **< 0.001** **0.007** 0.52 **0.003** N/A
Laboratory investigations				
Hemoglobin (g/dL), mean (SD) Anemia for age, % Platelet (x10^3^/cu mm), mean (SD) Thrombocytosis (> 450, 000/cu mm), % Erythrocyte sedimentation rate (mm/h), mean (SD) ≥ 20 mm/h, % C-reactive protein (mg/L), mean (SD) ≥5 mg/L, % Albumin (g/L), mean (SD) Hypoalbuminemia (< 35 g/L), %	10.1 (3.4) 74.7 514 (231) 66.2 49.5 (24.5) 87.3 36.9 (68.6) 74.7 33.1 (7.7) 49.3	9.7 (1.8) 74.3 526 (235) 68.6 51.1 (24.8) 88.6 47.3 (45.1) 94.3 30.4 (7.4) 62.9	10.5 (4.5) 75.0 506 (233) 63.9 47.8 (25.1) 86.1 28,0 (85.6) 55.6 35.5 (7.2) 36.1	0.33 0.95 0.72 0.68 0.58 0.76 0.24 **< 0.001** **0.004** **0.02**
Moderate to severe disease (defined by disease activity score^**†**^) at presentation, %	62.0	68.6	55.6	0.26

Abbreviations: CD, Crohn’s disease; IBD, inflammatory bowel disease; UC, ulcerative colitis. **Bold** numbers defined *P* value of < 0.05

*One child with IBD-unclassified. ^**†**^Disease severity defined by pediatric Crohn’s disease activity index (PCDAI) score for Crohn’s disease and pediatric ulcerative colitis activity index (PUCAI) for ulcerative colitis. Moderate to severe disease were defined when PCDAI > 30 for Crohn’s disease and PUCAI ≥ 35 for ulcerative colitis

### Clinical presentation and laboratory investigations

More than half (56.3%) of PIBD children in our cohort did not exhibit abdominal pain upon presentation, while diarrhea and bloody stool were most common symptoms. Perianal lesions were documented in 11/35 CD children (31.4%), of which 7 had large perianal skin tags, 3 had perianal abscess and 2 had perianal fistula. With regards to extraintestinal manifestations (EIMs), among 35 CD children, oral ulcers were documented in 8 children (23%), joint pain in 4 (11%), and erythema nodosum in 1 child (3%). Three cases with primary sclerosing cholangitis and three cases with autoimmune hepatitis were noted only in the UC children (6/36, 16.6%), and none of CD children had autoimmune liver disease.

Laboratory investigations at diagnosis revealed that 74.7% had anemia with a mean hemoglobin of 10.1 g/dL (SD 3.4), and 66.2% with thrombocytosis. High ESR and CRP was noted in 87% and 75%, respectively. Only 7 children (9.7%) had both normal ESR and CRP at diagnosis. A significantly higher proportion of children with CD had abnormal CRP and hypoalbuminemia when compared to UC (94.3% vs. 55.6%, *P* < .001; and 62.9% vs. 36.1%, *P* = 0.004, respectively) (Table 1). As shown in Table 2 which compares the characteristics of VEO-IBD and non-VEO-IBD children, no significant differences are noted with regards to baseline data, initial clinical manifestations, laboratory parameters, or therapies used between the two groups.

**Table 2:** Features of IBD children classified by the very early onset IBD status

Characteristics	Very early onset IBD		*P* value
	Yes, *n* = 27	No, *n* = 44	
CD vs. UC	14:13	21:23	0.74
Female, %	25.9	47.7	0.07
Male to female ratio	2.86:1	1.10:1	
Duration from onset to diagnosis, mean (SD), months	7.7 (8.0)	10.8 (15.9)	0.34
Presenting symptoms and signs, %			
Diarrhea Bloody stools Fever Weight loss/failure to thrive	66.7 81.5 22.2 55.6	70.5 65.9 31.8 47.7	0.74 0.16 0.38 0.52
Laboratory investigations			
Anemia for age, % Thrombocytosis (> 450, 000/cu mm), % C-reactive protein ≥ 5 mg/L, % Hypoalbuminemia (< 35 g/L), %	81.5 63.0 74.1 44.4	70.5 68.2 75.0 52.3	0.30 0.65 0.93 0.52
Moderate to severe disease (defined by disease activity score^*^) at presentation, %	70.4	56.8	0.25
Corticosteroid use during the first 3 months of diagnosis Early immunomodulator use	59.3 51.8	68.1 56.8	0.45 0.68

Abbreviations: CD, Crohn’s disease; IBD, inflammatory bowel disease; UC, ulcerative colitis. One child in this cohort was diagnosed with IBD-unclassified. * Disease severity defined by pediatric Crohn’s disease activity index (PCDAI) score for Crohn’s disease and pediatric ulcerative colitis activity index (PUCAI) for ulcerative colitis. Moderate to severe disease were defined when PCDAI > 30 for Crohn’s disease and PUCAI ≥ 35 for ulcerative colitis

### Disease severity, location, and phenotypes of IBD

Children in our cohort initially presented with a moderate-severe disease both in CD (68.6% with PCDAI > 30) and in UC (55.6% with PUCAI ≥ 35) without a significant difference between CD vs. UC; however, most UC children had never been defined as having a severe disease with PUCAI ≥ 65 points (88.9% with S0). In CD patients, two most common disease locations were colonic (L2) and ileocolonic (L3) (40.0% and 31.4%, respectively), and L4 involvement in 28.6%, with inflammatory (B1 phenotype in 88.5%) followed by penetrating (B3) in 8.6% and stricturing disease (B2) in 2.9%. Pancolonic involvement was the most involved location in UC (E4 phenotype in 88.9%).

At diagnosis, magnetic resonance enterography (MRE) was performed in 9 children and abdominal CT was performed in 12 children. None had CT enterography. The most common findings were small bowel wall thickening in 10 (seven with L4a phenotype and three with L4b phenotype among CD children), colonic wall thickening in 3 children, and 1 child had documented perianal fistula on MRE. Among 15 GI contrast studies, 4 had small bowel wall thickening (unspecified location) and 1 child had documented enteroenteric fistula. One child had video capsule endoscopy performed and demonstrated villous atrophy in the proximal jejunum and multiple ulcers in the distal ileum.

### Treatment and 1-year follow up after the PIBD diagnosis

During the first 3 months after PIBD diagnosis, 64.8% were given at least one form of systemic corticosteroids to induce remission (Table 3). No formal exclusive enteral nutrition was prescribed during the study period. All except three (91.7%) UC patients received 5-aminosalicylic acid (5-ASA), while CD patients received 5-ASA in 37.1%. Two UC children received azathioprine during induction with corticosteroids without 5-ASA, and another UC child received up-front infliximab due to a severe disease. On the other hand, 80% of CD children received azathioprine early in the disease course as compared to 30.6% (*P* < 0.001 both for 5-ASA and azathioprine). Only 8 children (3/8 in the VEO-IBD group and 6/8 in CD children) received infliximab during the first 3 months after diagnosis and 5 children had a 1-year follow-up data.

**Table 3:** Treatment during the first 3 months of IBD diagnosis and at 1 year after the diagnosis

Medications (%)	At diagnosis (*n* = 72)	CD (*n* = 35)	UC (*n* = 36)	*P* value (CD vs. UC)	1 year after diagnosis (*n* = 62)	CD (*n* = 28)	UC (*n* = 34)	*P* value (CD vs. UC)
Corticosteroids	64.8	82.9	47.2	0.002	43.7	51.4	36.1	0.19
5-aminosalicylic acid	64.8	37.1	91.7	< 0.001	47.9	22.9	72.2	< 0.001
Immunomodulators/calcineurin inhibitors^*^	55.6	82.6	30.6	< 0.001	51.6	78.6	29.4	< 0.001
Infliximab	11.3	17.1	5.6	0.12	15.5	17.1	13.9	0.71
**Others, n**								
Adalimumab		1				1		
Budesonide						1	1^**^	
Metronidazole		2						
Ciprofloxacin		2						
Vancomycin		1	2				2	

Abbreviations: CD, Crohn’s disease; IBD, inflammatory bowel disease; UC, ulcerative colitis. One child in this cohort was diagnosed with IBD-unclassified.

^*^Immunomodulators/calcineurin inhibitors include azathioprine, 6-mercaptopurine, cyclosporin, and methotrexate

^**^Budesonide was tried in one UC child with steroid-dependent who was already on azathioprine but had severe reaction to infliximab

At 1 year after diagnosis (*n* = 62 with available clinical data, 86% follow up), failure to enter one-year clinical remission (i.e., PCDAI or PUCAI > 10) was documented in 19/62 children (30.6%) with 35.7% in CD and 26.5% in UC children (*P* = 0.49). With regards to flares, 28 episodes of flare in 20/62 (32.2%) patients (approximately 1.4 episodes per patient, ranged 1–4 times) were documented. We noted 29 hospital admissions (20 with flares, 6 with infections, 2 for abdominal pain and 1 for abscess drainage) in 18 patients (1.6 admissions per patient). Among the 38/62 (61.3%) who underwent repeated endoscopy at 1 year from the initial PIBD diagnosis, 12/38 (30%) had documented mucosal healing (MH). Stratifying by clinical remission status, among 16 patients who failed to enter clinical remission and had follow up endoscopy, only 3 patients (18.8%) had MH; while 9/22 patients (40.9%) who entered clinical remission also had MH (*P* < 0.05). The three patients who failed to enter clinical remission but had documented MH received escalated treatment before endoscopy.

We found that 43.7% remained on oral corticosteroids (51.4% in CD vs. 36.1% in UC, *P* = 0.19). When stratifying by clinical remission status, corticosteroid use at 1 year was documented in 14/19 children (74%) who failed to enter remission vs. 16/43 (37%) who successfully entered remission (*P* = .008). Two surgical procedures had been performed: one child underwent right hemicolectomy with terminal ileal resection and another one had perianal abscess drained; which both were diagnosed with CD. No death was reported.

### Predictors of failure to enter clinical remission and predictors of corticosteroid use

Multivariate analyses were performed by multiple logistic regression to define independent predictors of failure to enter 1-year clinical remission (Table 4). Univariable analysis showed several predictors including diarrhea, weight loss/failure to thrive, hypoalbuminemia, and moderate to severe disease; but two significant independent predictors of failure to enter clinical remission remained: diarrhea (OR 9.32, 95%CI: 1.05, 82.76) and weight loss/failure to thrive (OR 4.92, 95%CI: 1.24, 19.49) at presentation.

**Table 4:** Predictors of failure to enter clinical remission at 1 year after IBD diagnosis (*n* = 62)

Characteristics at diagnosis	Univariate analysis		Multivariate analysis	
	OR (95%Cl)	*P* value	OR (95%Cl)	*P* value
Age	0.90 (0.78, 1.04)	0.15		
Female	0.74 (0.24, 2.23)	0.59		
Duration from onset to diagnosis	1.02 (0.98, 1.05)	0.37		
Type of IBD (Crohn’s disease vs. ulcerative colitis)	1.54 (0.52, 4.55)	0.43		
Abdominal pain	1.11 (0.37, 3.33)	0.85		
Diarrhea	12.96 (1.58, 106.1)	0.02	9.32 (1.05, 82.76)	0.045
Weight loss/failure to thrive	6.33 (1.79, 22.41)	0.004	4.92 (1.24, 19.49)	0.02
Anemia	4.10 (0.83, 20.28)	0.08		
Hypoalbuminemia	4.73 (1.43, 15.59)	0.01		
Erythrocyte sedimentation rate > 20 mm/h	0.55 (0.11, 2.73)	0.46		
C-reactive protein > 5 mg/L	1.63 (0.45, 5.85)	0.46		
Moderate to severe disease (defined by disease activity score*)	4.64 (1.17, 18.27)	0.03		
Corticosteroid use	2.45 (0.69, 8.65)	0.16		
Immunomodulator use	1.40 (0.48, 4.15)	0.54		
Infliximab use	1.52 (0.58, 5.22)	0.44		

Abbreviations: IBD, inflammatory bowel disease. * Disease severity defined by pediatric Crohn’s disease activity index (PCDAI) score for Crohn’s disease and pediatric ulcerative colitis activity index (PUCAI) for ulcerative colitis. Moderate to severe disease were defined when PCDAI > 30 for Crohn’s disease and PUCAI ≥ 35 for ulcerative colitis

For corticosteroid use at 1 year after diagnosis (Table 5), two predictors showed a trend of significance in the univariable analysis (*P* = 0.08) and remained significant in the multivariable analysis (via forward stepwise selection and logistic regression analyses) which were female (vs. male) (OR 3.08, 95%CI: 1.02, 9.33) and CD (vs. UC) (OR 3.03, 95%CI: 1.02, 9.09). No interaction was found in the two aforementioned multivariable analyses.

**Table 5:** Predictors of corticosteroid use at 1 year after IBD diagnosis (*n* = 62)

Characteristics at diagnosis	Univariate analysis		Multivariate analysis	
	OR (95%Cl)	*P*	OR (95%Cl)	*P*
Age	1.01 (0.90, 1.13)	0.87		
Female	2.51 (0.89, 7.08)	0.08	3.08 (1.02, 9.33)	0.046
Duration from onset to diagnosis	0.99 (0.95, 1.02)	0.51		
Type of IBD (Crohn’s disease vs. ulcerative colitis)	2.50 (0.89, 7.14)	0.08	3.03 (1.02, 9.09)	0.046
Abdominal pain	0.74 (0.27, 2.06)	0.57		
Diarrhea	1.06 (0.36, 3.13)	0.92		
Weight loss/failure to thrive	2.19 (0.79, 6.05)	0.13		
Anemia	0.65 (0.21, 2.05)	0.47		
Thrombocytosis	1.20 (0.42, 3.41)	0.73		
Hypoalbuminemia	1.91 (0.70, 5.25)	0.21		
Erythrocyte sedimentation rate > 20 mm/h	1.29 (0.26, 6.29)	0.76		
C-reactive protein > 5 mg/L	2.10 (0.67, 6.65)	0.21		
Moderate to severe disease (defined by disease activity score*)	1.37 (0.49, 3.86)	0.55		

Abbreviations: IBD, inflammatory bowel disease. * Disease severity defined by pediatric Crohn’s disease activity index (PCDAI) score for Crohn’s disease and pediatric ulcerative colitis activity index (PUCAI) for ulcerative colitis. Moderate to severe disease were defined when PCDAI > 30 for Crohn’s disease and PUCAI ≥ 35 for ulcerative colitis

## Discussion

This multicenter national study reports several points in PIBD that may differ from the existing literature. Among the 72 included patients, we found that the mean age at diagnosis was 7.9 years with 38% being with VEO-IBD. The age at diagnosis in our cohort was younger when compared to previous studies from EUROKIDS (12.5 years), Korea (13.6 years), and Singapore (10.5 years) [5, 14, 15]. Epidemiologic studies from Korea demonstrated an increasing trend in incident cases with decreasing age at diagnosis [16, 17]. A recent multinational Asian PIBD cohort in 2022 reported a younger age at diagnosis (mean age of 9 years) with 29.3% VEO-IBD [4] The differences in the age at diagnosis may be caused by the following: (1) varied genetic predisposition either with ethnic difference or potentially monogenic IBD, and (2) early life environmental triggers such as childhood acute infectious gastroenteritis and/or antibiotics use [18]. We noted a male predominance in the overall PIBD and more significance in CD, similar to the previous reports [15, 16]. 

With regards to the presenting characteristics, only 44% exhibited abdominal pain, which may be due to the fact that these children were younger at diagnosis. A study from India that included 197 patients aged ≤ 14 years also reported that children with VEO-UC (< 6 years) had a lower proportion of expressed abdominal pain when compared to the older UC ones [19]. UC children were more commonly presented with bloody stools when compared to CD, while CD had several presenting features that define more pronounced systemic inflammation [20, 21]. Almost one-third of CD children in our cohort presented with perianal lesions. Anemia for age, thrombocytosis, elevated systemic inflammatory markers and hypoalbuminemia are the characteristic blood markers for PIBD [22]. A meta-analysis of individual patient data revealed that the aforementioned laboratory tests provided an area under the curve that can differentiate children with vs. without PIBD with an acceptable to excellent discriminative capacity (area under the curve of 0.76–0.84). However, the single best marker was fecal calprotectin (with an area under the curve of 0.95), [23]. Unfortunately, the test was not commonly performed in our country due to its unavailability in the past. Interestingly, we found that only a small proportion (9.7%) of PIBD children had normal ESR and CRP. A study from the UK revealed that among 256 PIBD children, 21.9% had normal ESR and CRP and 9% had all normal blood tests [21]. Therefore, even with normal inflammatory markers, clinicians should be aware of PIBD in young children presented with suggestive signs and symptoms and consider referral for GI endoscopy and biopsy to confirm the diagnosis. We could not observe the differences in the initial disease burden or severity when stratifying by the VEO-IBD status (Table 2). Conflicting with our result, a recent report from Canada demonstrated that the infantile-onset IBD had worse outcomes than the older children [24]. 

A large proportion (65%) of children in our cohort were given at least one form of systemic corticosteroids as an initial treatment with the aim to induce disease remission, which would likely be due to the fact that most children presented with moderate to severe disease both in CD (69%) and UC (56%). Huang et al. reported that Asian PIBD children initially presented with moderate to severe disease in 54% of CD and 49% of UC cases [4]. Another potential explanation was that exclusive enteral nutrition was not widely used during the study period therefore most CD children with mild to moderate disease severity likely received systemic corticosteroids early on after the PIBD diagnosis. Aminosalicylates and azathioprine were the mainstay maintenance medications for UC and CD children (91.7% and 82.6%), respectively (Table 3). A small proportion of children received infliximab during the first 3 months after diagnosis (17.1% in CD and 5.6% in UC) and at 1 year after diagnosis (17.1% in CD and 13.9% in UC). It was not until 2022 that the Thai government pays for the anti-TNF therapy via the universal health coverage program, therefore treatment with anti-TNF was limited during the study period due to its high cost. Recently, ECCO-ESPGHAN statement suggested an ‘up-front’ anti-TNF therapy in CD children with predictor(s) of poor outcome such as inflammatory (B1) phenotype with extensive disease involving small bowel and colon, B1 with perianal disease, B2 and/or B3 phenotypes [25]. This strategy would result in a higher rate of remission and better long-term outcome with less surgical requirement. However, the number of patients in our cohort who received infliximab was relatively small which limited the robust statistical analysis.

Among 62 children (86% follow-up) with available data at 1 year after diagnosis, almost one-third failed to enter clinical remission with a non-significant difference between CD and UC. Among those who failed to enter clinical remission and underwent a follow-up endoscopy, only 18.8% had documented MH when compared to 40.9% among children who successfully entered clinical remission at 1 year after diagnosis. We found several variables in the univariable analyses associated with failure to enter remission: diarrhea, weight issues, hypoalbuminemia, and moderate-severe disease. However, diarrhea (OR 9.3) and weight issues (OR 4.9) at the initial presentation remained significant independent predictors in the multivariable analyses. Several adult studies in UC have shown that frequent bowel movements and low albumin level predicted treatment failure, colectomy [26], and greater usage of immunosuppression [27]. Growth failure is considered a poor prognostic outcome in PIBD especially among CD children therefore these patients may have a lower capacity to achieve clinical remission.

Almost half of the patients remained on corticosteroid at 1-year after diagnosis. Two predictors showed a trend of significance in the univariable analysis but became significant in the multivariable analysis (females [OR 3.1] and CD [OR 3.0]) (Table 5). Gender difference in the use of steroids is difficult to explain by biological plausibility. In contrast, an adult study found that UC females had a higher remission rate and lower colectomy rate at 1 year when compared to males [28], the results may imply some differences as compared to our study. CD initially had a higher proportion of children who receive corticosteroids from the beginning when compared to UC which may explain the finding of significance at a 1-year follow up. However, a 19% drop in the use of 5-ASA was noted in the UC children. We noted that 8 patients had 5-ASA discontinued and 5/8 possibly had intolerance. We therefore hypothesized that some proportion of these children may suffer from 5-ASA intolerance which has been previously reported in 13.8% [29]. As previously mentioned that only 15.5% received infliximab at 1 year after diagnosis, these children may not have their disease under good control with highly efficacious maintenance medications such as anti-TNF therapy hence leading to the need of systemic corticosteroids.

We are aware of some limitations in our study. Most data were retrospectively collected and may subject to missing information. We did not routinely perform genetic and/or immunologic testing in all VEO-IBD cases during the earlier years. The information on small bowel evaluation in our cohort were limited therefore the finding of disease location for CD may be different when compared to other studies. There was a limited number of patients received biologic agents and the ones with follow up fecal calprotectin and endoscopy to define MH (i.e., endoscopic remission) which is now considered as an optimal target of IBD management both in children and adults [30]. Both multivariable analyses also had limited number of patients to perform robust analyses especially during the forward stepwise selection of interested variables to make stable models, therefore only few strong predictors could be added into the final multivariable models.

## Conclusions

From this multicenter national study conducted in Thailand, PIBD presented earlier in life when compared to previous reports with 38% being VEO-IBD. CD was more likely to present with significant systemic inflammatory burden. Almost one-third of the patients in this cohort did not achieve clinical remission at 1 year after IBD diagnosis. Diarrhea and weight issues at diagnosis predicted a failure of entering 1-year clinical remission; while females and CD were at risk of corticosteroid use at 1 year after diagnosis.

## Data Availability

The data that support the findings of this study are available from the corresponding author, ST, upon reasonable request.

## Abbreviations

5-ASA: 5-aminosalicylic acid
CRP: C-reactive protein
CD: Crohn’s disease
EO-IBD: Early onset IBD
ESR: Erythrocyte sedimentation rate
GI: Gastrointestinal
IBD-U: Inflammatory bowel disease-unclassified
MH: Mucosal healing
PCDAI: Pediatric Crohn’s Disease Activity Index
PIBD: Pediatric inflammatory bowel disease
PUCAI: Pediatric Ulcerative Colitis Activity Index
UC: Ulcerative colitis
VEO-IBD: Very early onset IBD
